# Postpartum acute fatty liver of pregnancy: a case report

**DOI:** 10.1186/s13256-018-1593-3

**Published:** 2018-06-01

**Authors:** Naser Al-Husban, Oqba Al-Kuran, Amal Al Helou

**Affiliations:** Obstetrics & Gynecology Department, Faculty of Medicine, University of Jordan and Jordan University Hospital, P.O. Box 2194, Amman, 11941 Jordan

**Keywords:** Fatty liver, Pregnancy, Liver dysfunction, Postpartum, Skin rash

## Abstract

**Background:**

Acute fatty liver of pregnancy can be a very dramatic clinical event with significant risk of mortality to healthy women. The pathogenesis is still unknown. It usually occurs in the third trimester or in the immediate postpartum period. The clinical presentation is very variable. Medical staff have to be very cautious even regarding a minor complaint of feeling unwell. Skin rash has not been reported as one of the initial presentations of acute fatty liver of pregnancy. It is best treated in a center with a multidisciplinary approach. Admission to the intensive care unit is recommended.

**Case presentation:**

We report a case of a 20-year-old Middle Eastern Arabic woman who developed an acute fatty liver of pregnancy. She was not known to have any medical disease. She had had two previous uncomplicated deliveries. She developed acute fatty liver of pregnancy on the first day after an uncomplicated normal vaginal delivery of a healthy male newborn. She started to have nonitchy skin rash over her abdomen and upper limbs. Then she started to feel unwell. Twelve hours later, she developed epigastric and right upper quadrant abdominal pain, followed by jaundice, nausea, and vomiting. She developed recurrent hypoglycemic attacks, hemolytic anemia, coagulopathy, and hepatorenal syndrome.

**Conclusions:**

The clinical presentation of acute fatty liver of pregnancy is very variable and nonspecific. Skin rash can be a new presenting symptom of acute fatty liver of pregnancy. Immediate suspicion of the diagnosis, appropriate investigations, and urgent initiation of therapy in an intensive care unit and by a multidisciplinary team resulted in a good outcome with no adverse health consequences for our patient.

## Background

In 1940, Sheehan first recognized acute fatty liver of pregnancy (AFLP) as a distinct clinical syndrome and reported a series of six cases from the Glasgow Royal Maternity Hospital [1]. AFLP can be a very dramatic clinical event with sudden and catastrophic consequences to healthy women. It remains a disease of unknown etiology and pathogenesis [2]. This serious condition usually occurs in the third trimester or in the immediate postpartum period [3]. There exists some medical evidence to suggest that AFLP may be due to disordered metabolism of fatty acids in the maternal mitochondria [4]. It is best treated in a center with expertise in high-risk obstetrics, maternal-fetal medicine, neonatology, and hepatology. Experts in liver transplantation may be needed in severe cases. Admission to the intensive care unit (ICU) is recommended [3]. In our literature research, we found no cases of AFLP reported to present initially as skin rash.

## Case presentation

Our patient was a 20-year-old Arabic Middle Eastern woman. She was not known to have any medical illness. She had had two previous uneventful pregnancies with uncomplicated vaginal deliveries. Her only antenatal visit to our hospital was at 38 weeks of gestation, when she presented in early labor. Her general physical examination was unremarkable. An ultrasound (US) scan showed a cephalic, normally grown fetus with decreased amniotic fluid. The patient’s whole blood platelet count was 182 × 10^9^/L, white blood cell count (WBC) was 11 × 10^9^/L, and whole blood hemoglobin (Hb) was 116 g/L. Her blood group was AB positive.

On vaginal examination, she was found to have a 3-cm dilated, 80% effaced cervix and intact membranes. She was augmented with artificial rupture of her membranes and syntocinon intravenous infusion. Six hours later, she had an uneventful vaginal delivery of a healthy male newborn weighing 3.06 kg. The baby’s Apgar scores at 1 and 5 minutes were 8 and 9, respectively.

**Fig. 1:**
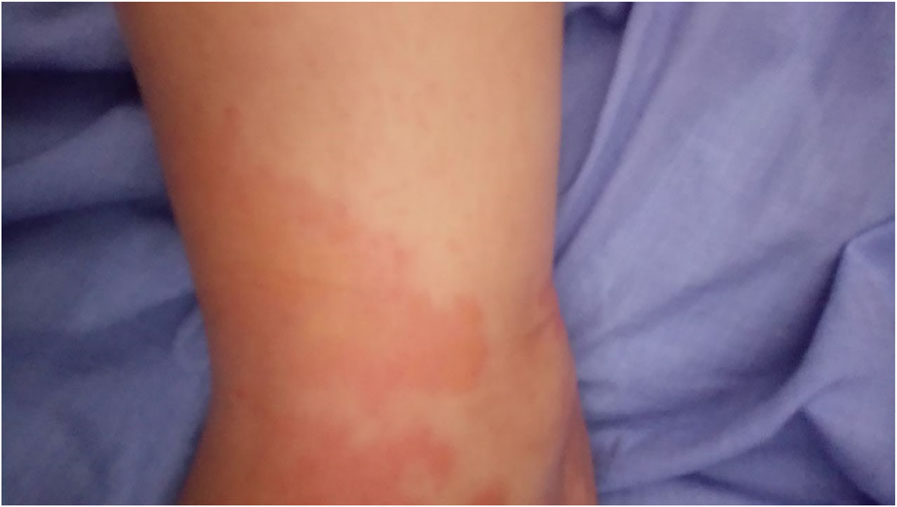
Skin rash over the right hand and forearm

**Fig. 2:**
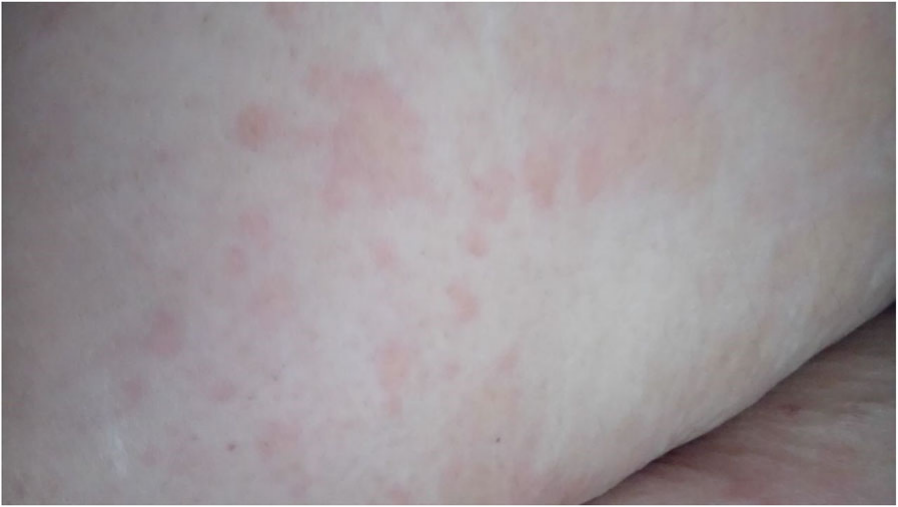
Skin rash over the abdomen

On the morning of her first postpartum day, the patient complained of a nonpruritic maculopapular skin rash over her upper limbs (Fig. 1), abdomen (Fig. 2), and back. It appeared suddenly as patchy lesions. It was not associated with pustules or vesicles. Her neck, face, and the palmar aspects of her hands and lower limbs were spared. There were no noticeable striae over her abdomen. She was not known to have any allergic reactions, and she did not receive any medications that could explain the findings. Twelve hours later, she was feeling very unwell and tired. She then developed generalized abdominal pain that increased in severity and was associated with nausea and occasional vomiting. Her vital signs were normal (blood pressure [BP] 120/70 mmHg, pulse rate 83 beats/minute, and oral temperature 37.1 °C). Her urine was yellow and turbid with 3+ proteinuria, and she had numerous WBC/high-power field (HPF) but no glycosuria. The same result was confirmed by testing a second urine sample that was obtained via a Foley catheter. A dermatologist’s review indicated nonspecific maculopapular skin rash, and the dermatologist advised only observation with no specific therapy but to investigate further. This advice alerted the medical staff to do further testing, which showed that her liver function, kidney function, whole blood count, serum glucose, serum lactate dehydrogenase (LDH), and coagulation profile were within normal limits.

Day 2 postpartum was marked by persistence of nausea and vomiting and a decrease in the intensity of skin rash. On day 3 postpartum, she had nausea, vomiting, and abdominal pain. Her skin rash showed a further decrease in intensity. She was very sick, pale, and jaundiced with epigastric and right upper quadrant abdominal tenderness. Her vital signs were stable. Investigations were repeated and showed thrombocytopenia (platelet count 54 × 10^9^/L), hypoglycemia (serum glucose 2.11 mmol/L), renal impairment (serum creatinine 228.75 μmol/L), impaired liver function (serum alanine aminotransferase [ALT] 0.735 μkat/L, serum aspartate aminotransferase [AST] 1.15 μkat/L, serum LDH 19.8 μkat/L, serum total bilirubin 68.4 μmol/L, serum direct bilirubin 58.15 μmol/L), and coagulopathy (plasma prothrombin time [PT] 22 seconds, control 14 seconds, blood partial thromboplastin time [PTT] 36 seconds, control 26 seconds, international normalized ratio [INR] 1.85) with normal urinalysis and normal plasma d-dimer and fibrin degradation products.

Acute fatty liver was suspected, and the patient was admitted to the ICU in the evening. In the ICU, her blood Hb was 88 g/L (dropped from 105 g/L), and her blood platelet count was 51 × 10^9^/L. Internist, hematologist, and anesthetist consultants were involved in her care. Septic workup was done, including urine and blood cultures, as well as high vaginal and endocervical swabs for culture and sensitivity. Because she was critically ill in the ICU with too many intravenous catheters and an indwelling urinary catheter, and because patients with AFLP are at risk of infection, a decision was taken by the multidisciplinary team to start her on a renal dose of imipenem/cilastatin. She was kept on intravenous fluid, normal saline (N/S) 100 ml/hour, and dextrose infusion. Five units of fresh frozen plasma (FFP), 5 U of cryoprecipitate, and 2 U of packed red blood cells (PRBCs) were given.

On the fourth day postpartum, the patient had persistent nausea, vomiting, and epigastric and right upper quadrant abdominal pain. Her vital signs were stable. She was jaundiced. Her skin rash had significantly decreased in distribution and intensity. She had a strict fluid input-output observation. Her urine output remained around 45–60 ml/hour. Her investigations showed anemia and thrombocytopenia (blood Hb 79 g/L and blood platelet count 44 × 10^9^/L), acute renal impairment (serum creatinine 316.4 μmol/L), very high serum LDH (19.7 μkat/L), elevated serum ALT (0.77 μkat/L), and elevated serum AST (1.52 μkat/L) with elevated serum direct and total bilirubin. Her serum glucose was 3.38 mmol/L (on dextrose infusion), and her total serum bile acids level was normal (6 μmol/L). Blood film showed hypochromic microcytic anemia, few schistocytes and acanthocytes, neutrophilia with toxic granulation of neutrophils, a majority of neutrophils that were hypersegmented, and thrombocytopenia. She received 2 U of PRBCs, 2 U of FFP, and 4 U of cryoprecipitate and was started on dexamethasone 4 mg intravenously every 8 hours.

In the afternoon, after transfusion of blood and blood products, her blood platelet count was 38 × 10^9^/L, blood Hb 97 g/L, and blood WBC 14.9 × 10^9^/L. Other tests revealed plasma PT 17.5 seconds, blood PTT 29.7 seconds, and INR 1.4 (corrected by the infusion of the blood and blood products).

An abdominopelvic computed tomographic (CT) scan without contrast enhancement revealed only hyperdense free fluid (ascites). A chest x-ray (CXR) showed congestive pulmonary changes and blunted bilateral costophrenic angles. She was started on furosemide 20 mg intravenously every 4 hours, intravenous fluid dextrose 25% 50 ml/hour, and N/S 0.9% 50 ml/hour.

On the fifth day postpartum (the third day in the ICU), the patient still felt unwell with epigastric and right upper quadrant abdominal pain and recurrent attacks of hypoglycemia. She had no skin rash at all. She had normal BP readings with mild epigastric and right upper quadrant tenderness. Her laboratory tests showed anemia, thrombocytopenia, hypoglycemia, leukocytosis, renal impairment, hyperbilirubinemia, and elevated serum LDH. Urinalysis showed 1+ proteinuria and hematuria. The result of a viral hepatitis screen was negative.

An abdominal U/S scan showed a marked amount of free fluid in the abdomen, liver span 17 cm, spleen span 14 cm, and a normal hepatobiliary tree with no stones or dilatation. A CXR was normal. She was given 5 U of FFP and kept on the antibiotic because of the ascitic fluid.

On the sixth day postpartum (fourth day in the ICU), the patient showed significant clinical improvement with stable vital signs (V/S). Her blood tests showed persistent anemia, thrombocytopenia, leukocytosis, elevated serum creatinine, elevated serum LDH, mild elevation of serum bilirubin, normal serum glucose, and normal liver enzymes and coagulation. A repeat blood film showed hypochromic microcytic anemia with mild anisocytosis, neutrophilic leukocytosis, few hypersegmented neutrophils and thrombocytopenia with large forms. She was prophylactically given 5 U of FFP as suggested by the multidisciplinary team.

On the seventh day postpartum (fifth day in the ICU), the patient started to show much clinical improvement (very mild nausea, occasional vomiting, and mild abdominal pain) with stable V/S. Blood tests showed Hb 98 g/L, blood platelet count 60 × 10^9^/L, blood WBC 16 × 10^9^/L (76% neutrophils and 16% lymphocytes), serum glucose 6.1 mmol/L, serum creatinine 251.6 μmol/L, serum urea nitrogen 52.1 mmol/L, and serum LDH 11.6 μkat/L with normal electrolytes and liver enzymes.

A CXR showed reticular shadowing bilaterally, a blunt left costophrenic angle, and a clear right costophrenic angle, which further supported the continuation of the antibiotic. She was given 4 U of FFP.

On the eighth day postpartum (the sixth day in the ICU), the patient was very well with no nausea, vomiting, or abdominal pain. Her dextrose infusion was disconnected. She was started on oral intake of fluids. She remained normoglycemic. She was prophylactically given 5 U of cryoprecipitate, 5 U of FFP, and 2 U of PRBCs for of her mild thrombocytopenia and anemia. In the evening, repeat blood test results were normal apart from mild elevation of serum creatinine. A decision was taken to discharge her from the ICU.

On the ninth day postpartum (the first day in the obstetric ward), the patient was very well with no complaints. She resumed breastfeeding in addition to artificial supplement. Her laboratory test results were normal. Her full septic workup result was negative. Imipenem/cilastatin was discontinued.

On the tenth day postpartum, the patient was very well and had no complaint. The results of her blood tests were normal apart from very mildly elevated serum creatinine.

The patient’s 11th postpartum day was unremarkable; she had no complaints and normal laboratory test results.

On the 12th day postpartum (4th day in the obstetric ward), the patient was very well with stable vital signs and no complaints. She had normal serum glucose, normal serum electrolytes, and normal liver enzymes and serum bilirubin (total and direct). Her serum LDH was 10.1 μkat/L, blood Hb 105 g/L, blood platelet count 584 × 10^9^/L, blood WBC 11.6 × 10^6^/L, and serum creatinine 1.43. In the afternoon, she was discharged to home receiving no medications.

The patient was seen in the clinic 1 week later. She was doing well with no complaints and was seeking contraception.

One month later, she and her baby were doing well with no complaints. In the clinic, she had an intrauterine contraceptive device inserted. The chronological order of her symptomatology and laboratory results are shown in Tables 1 and 2, respectively.

**Table 1:** Patient’s symptoms in chronological order postpartum

	Day 1	Day 2	Day 3	Day 4	Day 5	Day 6	Day 7	Day 8	Day 9	Day 12 (discharged to home)
Skin rash	+++	++	+	+	−	−	−	−	−	−
Nausea	+	+++	+++	+++	+	Very mild	Very mild	−	−	−
Vomiting	+	+++	+++	++	+	Occasional	Very occasional	−	−	−
General feeling	Very unwell	Very unwell	Extremely unwell	Unwell	Unwell	Well	Very well	Very well	Very well	Very well
Abdominal pain	+	+++	+++	++	+	Very mild	−	−	−	−

+ mild, ++ moderate, +++ severe, - no or nil

**Table 2:** Laboratory test results in chronological order postpartum

	Day 1	Day 2	Day 3	Day 4	Day 5	Day 6	Day 7	Day 8	Day 9	Day 10	Day 11	Day 12
Hb, g/L	112		88	79, then 97 after transfusion	94	98	98		101		102	105
Platelets, ×10^9^/L	161		54 then 51	44 then 38	30	34	60		80		482	584
WBC, ×10^9^/L	10.0		7.0	9.0 then 14.9	14.9	15.0	16.0		12.0		14.0	11.6
Serum creatinine, μmol/L	79.5		265.2	366.8	485.3	371.2	291.7	236.0	203.3	190.0	147.6	126.8
Serum urea nitrogen, mmol/L	10.7				56.8	55.7	52.1	50.7		40.7	33.5	
Serum ALT, μkat/L	0.27		0.735	0.77		0.42	N				N	
Serum AST, μkat/L	0.22		1.15	1.52		0.57	N				N	
Serum bilirubin direct, μmol/L	1.71		58.15	71.8	34.2	22.2					10.3	
Serum bilirubin total, μmol/L	5.13		68.4	82.1	42.8	30.8					15.4	
PT, seconds	12		22	17.5	14	14.5						
PTT, seconds	26		36	29.7	26	28						
INR	1.0		1.85	1.4	1.06	1.11			1.1			
Plasma d-dimer, nmol/L	1.9		N	9.8								
FDP, mg/L	7.0		N									
Plasma fibrinogen, μmol/L	9.0		N		3.36							
LDH, μkat/L	5.37		19.8	19.7	19.39	14.5	11.6				14.16	10.1
Urinalysis	3+ proteinuria, numerous WBC/HPF, no glycosuria		N	N	+ Proteinuria, 8–10 RBC/HPF							
Serum glucose, mmol/L	3.4		2.11	3.38	3.0	4.1	6.1	3.9	5.1	4.2	5.2	4.7

*Abbreviations: ALT* Alanine aminotransferase, *AST* Aspartate aminotransferase, *FDP* Fibrin degradation products, *Hb* Hemoglobin, *HPF* High-power field, *INR* International normalized ratio, *LDH* Lactate dehydrogenase, *mg/L* milligram/Litre, *mmol/L* millimole/Litre, *N* normal, *nmol/L* nanomole/Litre, *PT* Prothrombin time, *PTT* Partial thromboplastin time, *RBC* Red blood cell, *μmol/L* micromole/Litre, *μkat/L* microkatal/Litre, *WBC* White blood cell, *g/L* gram/Litre, + 1 proteinuria

Values are given in standard international units

## Discussion

In our literature research, we did not come across skin rash preceding or being part of an AFLP presentation. Our patient’s skin rash was different from pruritic urticarial papules and plaques of pregnancy because it was neither pruritic nor associated with striae, and it involved both upper limbs and the abdomen [5].

Initially, the diagnosis of AFLP was suspected because of the abrupt onset of feeling very unwell; abrupt onset of abdominal pain, nausea, and vomiting; and the attacks of severe hypoglycemia. The usual presentation of AFLP is nonspecific [6]. The diagnosis of the condition is suggested by jaundice, mild liver enzyme elevation, elevated WBC, disseminated intravascular coagulation (DIC), and a clinically unwell patient [6]. All these features were very apparent and evident in our patient (raised serum bilirubin, raised blood WBC and DIC). The differential diagnosis includes preeclampsia, HELLP syndrome (hemolysis, elevated liver enzymes, and low platelets), viral hepatitis, and obstetric cholestasis [3, 6–8]. Our patient’s BP remained normal prior to delivery and all through her hospital stay until discharge. She had no itching to suggest obstetric cholestasis, and her serum bile acid level was normal (6 μmol/L). Her viral hepatitis screen result was negative.

At an early stage, these patients may have an upper gastrointestinal hemorrhage due to coagulation abnormalities, acute renal failure, infection, pancreatitis, or hypoglycemia [9, 10]. Our patient had acute renal failure and persistent hypoglycemia with the need for strict input-output observation and intravenous dextrose infusion. She maintained a normal urine output. The association of transient diabetes insipidus and AFLP appears more common than previously recognized. Both may be part of the spectrum of preeclampsia [11]. Hypoglycemia and prolongation of PT helped us to differentiate our patient’s presentation from HELLP syndrome. DIC is relatively common in these cases [12, 13]. Our patient received appropriate infusions of FFP and cryoprecipitate. She also developed thrombocytopenia, which is a known complication of AFLP [14]. We obtained both a CT scan and a US scan. Both imaging modalities are noninvasive but have limited usefulness in the AFLP diagnosis [15].

Our patient’s blood laboratory test results showed marked elevation of serum bilirubin and jaundice with only mild liver enzyme elevation. She also had leukocytosis and ascites. These results, in addition to the patient’s clinical symptoms and hypoglycemia, were consistent with the diagnosis of AFLP [16].

The patient went through hemostatic dysfunction in the form of hemolytic anemia and DIC, as indicated by the hematological test results. Hemostatic dysfunction started very early in her condition and persisted for a few days thereafter. In those patients who develop AFLP prior to delivery, this dysfunction persists 4–5 days postpartum [17]. Our patient received infusions of PRBCs, FFP, and cryoprecipitate. Severe cases of AFLP can lead to coagulopathy, liver failure, and hypoglycemia. The pathological hepatic condition is usually self-limiting, with liver function returning to normal 7–9 days after delivery [18, 19]. Fluid therapy in our patient was very strict to avoid pulmonary edema caused by low plasma oncotic pressure.

In the ICU, our patient was conscious, alert, and did not need ventilator support. Patients who have received ventilator support or encephalopathy and failed to respond to conventional supportive therapy have benefited from plasma exchange alone or in combination with continuous hemodiafiltration [20–22]. We started our patient on an antibiotic and watched her carefully for the development of any sign of adult respiratory distress syndrome (ARDS). On the basis of her CXR, she needed an intravenous diuretic. ARDS might occur as a complication of acute liver failure, septicemia, or transfusion of multiple blood products [23].

After the fifth day of the clinical onset of our patient’s presentation, she started to show clinical and hematobiochemical improvement. In patients who develop AFLP antenatally, clinical recovery typically is seen within 3–4 days; however, laboratory abnormalities can persist much longer [24].

Our patient made a quick, uncomplicated recovery. We found a case report of massive intrahepatic calcification [25]. AFLP can progress to fulminant hepatic failure with the need for liver transplant, encephalopathy, coma, and death [26, 27]. The clinical manifestations of patients with mutations in enzymes of fatty acid metabolism may include AFLP that may mimic severe preeclampsia [28].

## Conclusions

The clinical presentation of AFLP is very variable and nonspecific. Skin rash can be a new presenting symptom of AFLP. In our patient, immediate suspicion of the diagnosis, appropriate investigations, and urgent initiation of therapy in an ICU and by a multidisciplinary team resulted in a good outcome with no adverse health consequences.

## Abbreviations

AFLP: Acute fatty liver of pregnancy
ALT: Alanine aminotransferase
ARDS: Adult respiratory distress syndrome
AST: Aspartate aminotransferase
BP: Blood pressure
CT: Computed tomographic
CXR: Chest x-ray
DIC: Disseminated intravascular coagulation
FDP: Fibrin degradation product
FFP: Fresh frozen plasma
Hb: Hemoglobin
HELLP syndrome: Hemolysis, elevated liver enzymes, and low platelets
HPF: High-power field
ICU: Intensive care unit
INR: International normalized ratio
LDH: Lactate dehydrogenase
N/S: Normal saline
PRBC: Packed red blood cell
PT: Prothrombin time
PTT: Partial thromboplastin time
RBC: Red blood cell
US: Ultrasound
WBC: White blood cell count

## References

[1] Sheehan HL. The pathology of acute yellow atrophy and delayed chloroform poisoning. J Obstet Gynaecol. 1940;47:49–61.

[2] Riely CA. Acute fatty liver of pregnancy. Semin Liver Dis. 1987;7(1):47–54. 10.1055/s-2008-1040563 3296215

[3] Ko H, Yoshida EM. Acute fatty liver of pregnancy. Can J Gastroenterol. 2006;20(1):25–30. 10.1155/2006/638131 PMC2538964 16432556

[4] Bellig LL. Maternal acute fatty liver of pregnancy and the associated risk for long-chain 3-hydroxyacyl-coenzyme a dehydrogenase (LCHAD) deficiency in infants. Adv Neonatal Care. 2004;4(1):26–32. 10.1016/j.adnc.2003.12.001 14988877

[5] Dehdashti AL, Wikas SM. Pruritic urticarial papules and plaques of pregnancy occurring postpartum. Cutis. 2015;95(6):344–7. 26125211

[6] Wei Q, Zhang L, Liu X. Clinical diagnosis and treatment of acute fatty liver of pregnancy: a literature review and 11 new cases. J Obstet Gynaecol Res. 2010;36(4):751–6. 10.1111/j.1447-0756.2010.01242.x 20666940

[7] Riely CA. Liver disease in the pregnant patient. Am J Gastroenterol. 1999;94(7):1728–32. 10.1111/j.1572-0241.1999.01199.x 10406228

[8] Pang WW, Lei CH, Chang DP, Yang TF, Chung YT, Huang MH. Acute jaundice in pregnancy: acute fatty liver or acute viral hepatitis? Acta Anaesthesiol Sin. 1999;37(3):167–70. 10609353

[9] Kaplan MM. Acute fatty liver of pregnancy. N Engl J Med. 1985;313:367–70. 10.1056/NEJM198508083130606 3892299

[10] Vigil-De Gracia P, Lavergne JA. Acute fatty liver of pregnancy. Int J Gynaecol Obstet. 2001;72:193–5. 10.1016/s0020-7292(00)00370-2 11166756

[11] Kennedy S, Hall PM, Seymour AE, et al. Transient diabetes insipidus and acute fatty liver of pregnancy. Br J Obstet Gynaecol. 1994;101:387–91. 10.1111/j.1471-0528.1994.tb11909.x 8018608

[12] Holzbach RT. Acute fatty liver of pregnancy with disseminated intravascular coagulation. Obstet Gynecol. 1974;43:740–4. 4545029

[13] Cano RI, Delman MR, Pitchumoni CS, Lev R, Rosenthal WS. Acute fatty liver of pregnancy. Complication by disseminated intravascular coagulation. JAMA. 1975;231:159–61. 1172681

[14] Burroughs AK, Seong NH, Dojcinov DM, Scheuer PJ, Sherlock SV. Idiopathic acute fatty liver of pregnancy in 12 patients. Q J Med. 1982;51:481–97. 7156326

[15] Castro MA, Ouzounian JG, Colletti PM, Shaw KJ, Stein SM, Goodwin TM. Radiologic studies in acute fatty liver of pregnancy: a review of the literature and 19 new cases. J Reprod Med. 1996;41:839–43. 8951135

[16] Knight M, Nelson-Piercy C, Kurinczuk JJ, Spark P, Brocklehurst P. UK obstetric surveillance system: a prospective national study of acute fatty liver of pregnancy in the UK. Gut. 2008;57:951–6. 10.1136/gut.2008.148676 18332072

[17] Nelson DB, Yost NP, Cunningham FG. Hemostatic dysfunction with acute fatty liver of pregnancy. Obstet Gynecol. 2014;124(1):40–6. 10.1097/AOG.0000000000000296 24901264

[18] Bacq Y. Acute fatty liver of pregnancy. Semin Perinatol. 1998;22:134–40. 10.1016/s0146-0005(98)80045-1 9638907

[19] Castro MA, Fassett MJ, Reynolds TB, et al. Reversible peripartum liver failure: a new prospective on the diagnosis, treatment and cause of acute fatty liver of pregnancy, based on 28 consecutive cases. Am J Obstet Gynecol. 1999;181:389–95. 10.1016/s0002-9378(99)70567-3 10454689

[20] Martin JN Jr, Briery CM, Rose CH, et al. Postpartum plasma exchange as adjunctive therapy for severe acute fatty liver of pregnancy. J Clin Apher. 2008;23(4):138–43. 10.1002/jca.20168 18633996

[21] Jin F, Cao M, Bai Y, et al. Therapeutic effects of plasma exchange for the treatment of 39 patients with acute fatty liver of pregnancy. Discov Med. 2012;13(72):369–73. 22642918

[22] Chu YF, Mei M, Juan Z, et al. Effectiveness of combining plasma exchange with continuous hemodiafiltration on acute fatty liver of pregnancy complicated by multiple organ dysfunction. Artif Organs. 2012;36(6):530–4. 10.1111/j.1525-1594.2011.01424.x 22428588

[23] Kalpana S, Veena R, Geeta P, et al. Acute fatty liver of pregnancy: a case report of an uncommon disease. Indian J Crit Care Med. 2009;13(1):34–6. 10.4103/0972-5229.53115 PMC2772253 19881179

[24] Nelson DB, Yost NP, Cunningham FG. Acute fatty liver of pregnancy: clinical outcomes and expected duration of recovery. Am J Obstet Gynecol. 2013;209(5):456–7. 10.1016/j.ajog.2013.07.006 23860212

[25] Bhat KJ, Shovkat R, Samoon HJ. Postpartum Acute liver dysfunction: a case of acute fatty liver of pregnancy developing massive intrahepatic calcification. Gastroenterol Res. 2015;8(6):313–5. 10.14740/gr693w PMC5051032 27785315

[26] Bacq Y. Liver diseases unique to pregnancy: a 2010 update. Clin Res Hepatol Gastroenterol. 2011;35:182–93. 10.1016/j.clinre.2010.11.011 21310683

[27] Heneghan MA, Selzner M, Yoshida EM, Mullhaupt B. Pregnancy and sexual function in liver transplantation. J Hepatol. 2008;49(4):507–19. 10.1016/j.jhep.2008.07.011 18715668

[28] Iruretagoyena JI, Shah D. A case of severe preeclampsia leading to the diagnosis of de novo abnormal fatty acid metabolism and ACE gene deletion. J Obstet Gynaecol Can. 2010;32(7):695–7. 10.1016/s1701-2163(16)34575-3 20707960

